# Well-Being and Sexual Diversity in Higher Education: The Role of Mental Health, Optimism, Academic Performance, and Motivation in Portuguese Students

**DOI:** 10.3390/healthcare14030407

**Published:** 2026-02-05

**Authors:** José Alberto Ribeiro-Gonçalves, Diana Fernandes, Ester Câmara, Margarida Pocinho

**Affiliations:** 1 Department of Psychology, University of Madeira, 9020-105 Funchal, Portugal; ester.camara@staff.uma.pt (E.C.); mpocinho@staff.uma.pt (M.P.); 2 Center for Psychology of University of Porto (CPUP), Faculty of Psychology and Educational Sciences, University of Porto, 4200-135 Porto, Portugal; 3 William James Center for Research, Ispa—Instituto Universitário, 1149-041 Lisboa, Portugal; 4 University Research Center in Psychology (CUIP), University of Madeira, 9020-105 Funchal, Portugal; 5 Faculty of Arts and Humanities, University of Madeira, 9020-105 Funchal, Portugal; 2087414@student.uma.pt

**Keywords:** academic performance, higher education, LGB students, mental health, well-being

## Abstract

Introduction. Entering higher education represents a significant and challenging stage, marked by substantial changes that impact students’ well-being, mental health, and academic performance. These challenges can be particularly intensified for lesbian, gay, or bisexual (LGB) students, who are frequently subject to minority stress. In this context, few studies have focused on aspects associated with the well-being of these students. This study aimed to assess well-being, mental health, optimism, motivation, and academic performance according to students’ sexual orientation, as well as the main predictors of well-being in both groups. Method. The sample comprised 285 higher education Portuguese students, of whom 129 were heterosexual and 156 were LGB, aged between 18 and 69 years. Results. The results revealed that the LGB students showed significantly lower levels of optimism, but higher levels of academic performance and mental health, compared to heterosexual students. Among LGB students, optimism stood out as one of the main predictors of well-being, while mental health stood out among heterosexual students. Conclusions. The results highlight the need for more inclusive university environments and psychological interventions focused on promoting optimism to reduce the impact of minority stress and promote the well-being and academic retention of LGB students. More studies are needed that consider the diverse sexual orientations of university students.

## 1. Introduction

Well-being is based on individuals’ assessment of their own life, considering their values, needs, and feelings [1,2]. This variable has been extensively studied in recent decades as a key subjective indicator of health in various contexts, ranging from health to social contexts [3,4]. In the context of education, some studies have also shown the importance of well-being [5,6,7], associating higher levels of happiness and well-being with better social interactions, affective relationships, and the avoidance of isolation [8]. Further, higher levels of self-esteem, self-efficacy, and overall well-being are strongly associated with more positive attitudes towards the present and the future. They are linked to lower school absenteeism, better academic performance, and improved psychosocial adjustment [9]. Recent studies also demonstrate a direct relationship between well-being in higher education and important pedagogical elements, such as the quality of learning, adequate support, and a positive learning environment [10].

The significant growth of opportunities to join higher education, mainly since the 1990s, has led to increased reflection on the new demands due to the greater heterogeneity of individual, academic, or psychosocial characteristics and needs [11]. For many students, higher education is marked by several challenges [12]. It is a period of transformation at both personal and professional levels, encompassing cognitive, affective, social, and professional aspects [13]. One characteristic that has been significantly associated with the educational demands of higher education is the sexual orientation of students [14,15]. Adjusting to higher education becomes particularly complex when the student belongs to a social minority, such as lesbian, gay, or bisexual (LGB) people, who frequently face discrimination, invisibility, and exclusion in educational contexts [16,17]. Studies indicate that LGB students experience subtle and explicit forms of prejudice and exclusion that can negatively affect their mental health [18].

For many LGB students, the academic environment remains a space of discomfort and insecurity. Studies indicate that about a quarter of these students avoid spaces such as bathrooms, sports facilities, bars, or canteens, and at least one in six missed classes because they feel unsafe or uncomfortable [17]. Further, data from the European Union Agency for Fundamental Rights [19] revealed that in Europe, education is one of the areas where LGB people experience the most discrimination and violence. In Portugal, only one in ten LGB people of pre-university age, between 15 and 17 years old, are openly aware of their gender identity or sexual orientation; moreover, about two-thirds reported experiences of discrimination, and half were victims of bullying [16]. Despite this data, there is still a significant lack of studies focusing on the sexual orientation of higher education students, particularly studies comparing students from sexual minorities and heterosexuals, and especially those that integrate not only risk variables (e.g., mental health, risky sexual behavior) but also protective and positive variables (e.g., motivation, optimism).

In Portugal, significant progress has been made in promoting inclusive education, as evidenced by evolving policies and practices that aim to create more equitable and accessible educational environments [20]. However, despite the general guidelines from the Ministry of Education, there are still no specific documents guiding the inclusion of LGB students. Similarly, in legislative terms, Portugal stands out internationally for its commitment to protecting LGB people and combating discrimination based on gender identity [21]. These include the protection of young people through non-discrimination clauses, the legalization of same-sex marriage in 2010, the recognition of parental rights for LGB people in 2016, and the law on gender self-determination in school and other contexts, starting at age 16, in 2018 [21,22,23]. In the educational sphere, legislation has sought to combat discrimination based on sexual orientation and gender identity by regulating sex education in schools, stipulating that schools must adopt support and monitoring measures whenever necessary [24]. Despite these legislative advances, Portugal pointed out to the Committee of Ministers of the Council of Europe that many recommendations have not yet been fully implemented, especially in the educational context [24]. This creates a gap between legal progress and the well-being of LGB students in the educational context, creating vulnerabilities. Furthermore, most of the gains in rights that directly affect LGB students have occurred cumulatively very recently, starting in the last decade, and few studies have captured how these changes are influencing the educational experience of Portuguese LGB individuals [25]. According to Gato [26], many students report the existence of a hostile school climate, characterized by discrimination, bullying, harassment, and invisibility.

**Figure 1:**
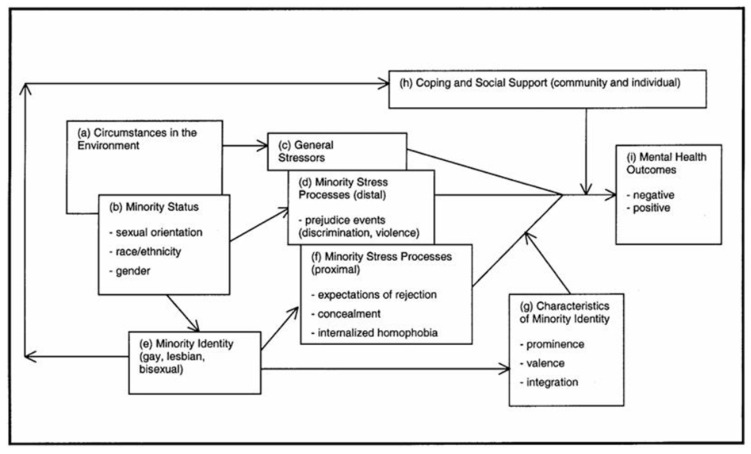
MSM—Minority Stress Process in the Lesbian, Gay, and Bisexual Population. Meyer (2003) [27]. Copyright© 2003, National Institute of Health.

The increased vulnerability of LGB students can be understood considering the Minority Stress Model (MSM [27]; Figure 1), which describes how specific stressors contribute to adverse psychological health outcomes [28]. This model establishes that young people from sexual minorities are exposed to increased levels of stress, compared to the heterosexual population, derived from their sexual identity (Minority Stress), including exposure to social and family rejection and the internalization of sexual stigma, as is the case in other LGB age groups [29,30,31]. According to Meyer [27], sexual stigma is closely linked to mental and physical health and compromised well-being in LGB youth; studies indicate that sexual stigma is associated with higher rates of depression, self-harm, and suicidal ideation in LGB youth compared to their heterosexual peers [32]. Further, these young people are at a greater risk of experiencing a lack of support from their family, the school community, and society as a whole [33]. As a possible consequence, studies reveal potential discrepancies in academic and health outcomes associated with negative school settings for LGB students, compared to heterosexual students [32,34]. Other studies have also highlighted that sexual stigma is directly related to increased anxiety and depression among LGB youth [35] and signs of anxiety in LGB people are often associated with shame, social avoidance, lack of family support, and discrimination [36].

It is also important to mention that the MSM recognizes and highlights the transformative potential of minority identity in this process. Thus, the results regarding the mental and physical health and well-being of these young people stem from the interaction between stress factors and protective factors. The latter include resources such as optimism and motivation [27,37], which have been suggested as important tools for coping with the consequences of sexual stigma in the school context. Optimism is considered a protective factor that contributes to greater resilience to stress, better adaptation to academic challenges, and greater persistence and motivation in adverse contexts. Studies indicate that students with higher levels of optimism tend to have better academic performance, greater subjective well-being [38], establish new interpersonal relationships, and explore the academic context with a positive outlook, relating to higher levels of well-being, engagement, and academic performance [39,40]. Longitudinal studies also demonstrate that motivation in LGB students may be intrinsically linked to school retention, as well as to a sense of belonging and academic identity, which in turn influence students’ well-being and academic performance [41].

Considering the importance of higher education promoting inclusive, safe, and equitable environments that favor the development of all students, regardless of their sexual orientation, this study aimed to evaluate the levels of well-being, mental health, and academic performance of LGB students and their heterosexual peers in the context of higher education, as well as the levels of protective variables, optimism, and motivation. In a second stage, it assessed which variables were found to be significant predictors of well-being levels in LGB and heterosexual students.

## 2. Methods

### 2.1. Participants

The study included 285 higher education students, self-identified as heterosexual (*n* = 129) or LGB (*n* = 156), aged 18 or older (see Table 1). Participants attended public and private educational institutions located in various regions of mainland Portugal and the islands. Two inclusion criteria were used: being at least 18 years old and being enrolled in a higher education institution in Portugal.

### 2.2. Instruments

This study employed six assessment instruments: a sociodemographic questionnaire, the Experiential Well-being Scale, the Optimism Scale, the Depression, Anxiety, and Stress Scale, a single-item measure of motivation, and a single-item measure related to academic performance.

#### 2.2.1. Sociodemographic Questionnaire

The sociodemographic questionnaire included questions about age, gender, marital status, area of residence, current study cycle, year of study, type of course being attended, and geographical area of study. Most questions were open-ended, with multiple-choice answers, except for the year and type of course attended, which were formulated as open-ended questions and later categorized.

#### 2.2.2. Experiential Wellbeing

The Experiential Well-being Scale, developed by Pocinho and Garcês [42], was used to assess variables associated with well-being from a positive psychology perspective. The scale has 10 items (e.g., Item 4: “I was able to see the positive side of the less pleasant situations that occurred”) presented on a 7-point Likert-type scale, ranging from 1—“Strongly disagree” to 7—“Strongly agree”. The total score ranges from 10 to 70 points. In the present study, the scale showed an acceptable internal consistency coefficient (α = 0.709).

#### 2.2.3. Optimism

The Optimism Scale, adapted for the Portuguese population by Barros [43], assesses general optimism and was used. This scale consists of 4 items (e.g., Item 4: “In general, I consider myself an optimistic person”), whose responses are evaluated using a 5-point Likert scale, ranging from 1—“strongly disagree” to 5—“strongly agree”. Data interpretation is performed by summing the values assigned to each item, which can range from 4 to 20 points. Regarding reliability, the scale showed high internal consistency (α = 0.893).

#### 2.2.4. Mental Health

The Depression, Anxiety and Stress Scale (DASS-21), adapted for the Portuguese population by Pais-Ribeiro, Honrado, and Leal [44], was used as an indicator of mental health. This instrument assesses symptoms associated with anxiety, depression, and stress, consisting of 21 items (e.g., Item 21: “I felt that life had no meaning”) organized into three subscales, each with seven items: the Anxiety subscale, the Depression subscale, and the Stress subscale. This scale uses a 4-point Likert-type response format, which assesses the frequency and intensity of symptoms in the last seven days, from 0—“does not apply to me at all” to 3—“applied to me most of the time”. For the interpretation of the results, the values of each subscale should be summed and then multiplied by two, resulting in an overall score that allows the symptomatology to be classified as an indicator of mental health. Higher scores indicated lower levels of mental health. The scale showed high internal consistency (α = 0.974).

#### 2.2.5. Academic Motivation

The following single item was used to measure academic motivation: “How motivated do you feel to go to university in your day-to-day life?” This type of measure, presented in a single-item format, has been successfully employed in previous research within the context of higher education [45]. This study aimed to gain insight into the motivation of university students regarding their pursuit of higher education. The item was answered using a 6-point Likert type scale, ranging from 0 (“Not motivated at all”) to 5 (“Very motivated”).

#### 2.2.6. Academic Performance

The following open-ended question was used to measure academic performance: “Indicate the overall average of the course units completed to date.” This question aimed to assess the overall average of course units completed up to the end of the first semester of the academic year 2024/2025. A self-report question, in open format, was chosen because it involves sensitive personal data, and to ensure the subjective perception of the participants, respecting their privacy and promoting greater adherence to the response. Empirical evidence from meta-analysis studies indicates that the overall average values obtained through self-reporting show a high correlation with official academic records [46], which supports its use as a reliable indicator of academic performance in similar contexts [46].

### 2.3. Procedures

We created an online questionnaire using Microsoft Forms. The researchers repeatedly tested this questionnaire to ensure its quality and functionality, and it was the primary means of data collection. After verifying the operational status of the data collection method, an exhaustive search was conducted for institutional contacts (emails of Rectors, Directors, and Communication Offices) from all universities located in mainland Portugal and the Autonomous Regions. Subsequently, partnerships were requested from the institutions to promote participation in the study. Additionally, the study was disseminated through electronic platforms, namely via institutional email (Outlook) addressed to youth institutions and associations, and promoted on social networks such as Facebook, Instagram, WhatsApp, X (Twitter), and LinkedIn.

The data collection period ran from November 2024 to February 2025. Participation was voluntary and preceded by a brief explanation of the study’s objectives. All procedures complied with the ethical standards of the Declaration of Helsinki and were approved by the institutional ethics committee from the University, with approval number [96/CEUMA/2024]. After data collection, analysis was performed using IBM SPSS Statistics (version 30). No imputation of missing data was made since the missing data for each variable never constituted more than 15% of the total responses (approximately 10% for LGB students and 11% for heterosexual students). Descriptive analyses were conducted, including Pearson bivariate correlations and t-tests for independent samples to compare means, as well as multiple linear regressions. All statistical assumptions necessary for these analyses were previously tested, and no violations were found, including in the normality analyses (|*Sk*| < 3 and |*Ku*| < 7). The linearity of the variables was verified using scatterplots, no problems were identified. The absence of multicollinearity was also verified in regressions (VIF < 2 in all independent variables). Results with *p* ≤ 0.05 were considered statistically significant, and *p <* 0.10 as marginally significant.

## 3. Results

Descriptive analysis revealed that students who identified as heterosexual (*n* = 129) had a slightly higher average age (average close to 28 years) compared to the average age (average close to 25 years) reported by LGB youth (*n* = 156). The age range of heterosexual participants (*min* = 18; *max* = 57) was slightly lower than that of LGB youth (*min* = 18; *max* = 69). The majority of participants were female, and almost three-quarters were from mainland Portugal. Nearly two-thirds of heterosexual people and almost three-quarters of LGB people were single, while more than half of the students in both groups were in the undergraduate degree. The sample was evenly distributed across all regions of the country, except for the inland. Finally, regarding academic fields, heterosexual students were primarily enrolled in health and life sciences courses, while LGB students predominated in social sciences, education, and arts (see Table 1).

It was found that, in both heterosexual and LGB students, well-being correlated very significantly (*p* < 0.01) and positively with the remaining variables, excluding the negative and significative correlation with mental health indicator, and except for academic performance in the heterosexual student group. In general, the remaining variables also exhibited significant and positive correlations with one another (see Table 2).

When assessing the average levels of the variables according to sexual orientation, it was found that heterosexual students presented higher levels of optimism, but lower academic performance and mental health compared to LGB students (see Table 3). Marginally significant differences (*p* < 0.10) were also observed between the groups regarding levels of well-being and motivation, with heterosexual students registering slightly higher values.

The regression model for heterosexual students was statistically significant, *F*(4, 115) = 19.891, *p* < 0.001, with an explained variance of 50.0% (adjusted *R*^2^ = 47.6%). The model indicates that well-being in this group was primarily predicted by lower levels of mental health, followed by higher levels of academic performance and optimism, and finally by higher levels of motivation. Regarding LGB students, the regression model was also statistically significant, *F*(4, 141) = 75.875; *p* < 0.001, with an explained variance of 63.2% (adjusted *R*^2^ = 62.6%). The model indicates that well-being in this group was primarily predicted by high levels of optimism, followed by lower levels of mental health. Higher levels of motivation also contributed to well-being, although to a lesser extent. In this group, academic performance levels were not a significant predictor of well-being (*p* = 0.334; See Table 4).

## 4. Discussion

The aim was to evaluate the levels of well-being, mental health, optimism, motivation, and academic performance in Portuguese university students according to their sexual orientation, and to verify how these latter variables predicted levels of well-being. In this sense, one of the main results was that LGB students presented higher levels of mental health and lower levels of optimism compared to heterosexual students. Regarding optimism, this result is not unexpected, several studies indicate that LGB students face greater challenges in the area of psychological health which can reduce your expectation of looking to the future in a positive way, namely higher levels of anxiety, depression, stress, and suicidal ideation, including studies within the framework of the minority stress model [27,33,47,48]. The need to conceal sexual orientation and the fear of rejection from peers and teachers can intensify feelings of isolation, shame, and inadequacy, compromising their ability to have hope for the future [49,50]. However, this result may primarily highlight that, facing the persistence of difficulties associated with experiencing sexual orientation in educational and other unsafe or unwelcoming contexts, the LGB students may be increasingly using resources to cope, where discrimination, prejudice, and the invisibility of identity are recurring experiences. Although partially contradicting the MSM, data such as these should be studied further as they may demonstrate the resilience developed by adversity in LGB students and probably a greater predisposition to seek help [25,51].

It was also found that LGB students achieved higher levels of academic performance than heterosexual students; this result is not in line with some research that associates minority status with worse academic results, associated to proximal and distal stress factors (see Figure 1) [34,52]. However, this result can be explained by compensation theory, which posits that people belonging to minority groups may engage more intensely in academic performance as a strategy for personal and social validation [53,54]. This behaviour can be interpreted as an adaptive response to minority stress, functioning simultaneously as a strategy for self-affirmation and a way to resist negative social expectations [27]. Some studies [55] argue that many LGB students transform adversity into motivation and optimism, thereby developing academic resilience and maintaining a commitment to their studies and academic success, even in hostile contexts. Despite this, we cannot ignore that this result may also be partly explained by some sampling bias or by inflation of responses due to self-reporting, and that academic performance was not a predictor of well-being in LGB students. It is also important to note that this result may be partly a product of programs recently implemented by the Ministry of Education to support academic success and integration, specifically programs aimed at preventing academic dropout and promoting mental health in higher education. Examples of these programs include: (1) IPC + Success 2.0 [56], (2) SoUAlg—Academic Success System of the University of Algarve [57], (3) +Success@UPortucalense [58], and (4) MindGuard-Mental Health in Higher Education [59].

Among heterosexual students, lower levels of mental health were the most significant predictor of well-being, while among LGB students, in addition to mental health, optimism stood out as a central factor. This distinction suggests that, although emotional and motivational components are crucial for all students, coping strategies and mobilised psychological resources may vary depending on the context and challenges experienced by each group [27,60]. Regarding heterosexual students, it is plausible that mental health represents a basic condition of functioning, with a direct impact on the perception of well-being. The absence of psychological symptoms such as anxiety or stress can translate into greater emotional stability, a sense of control, and satisfaction with university life. In the case of LGB students, although lower levels of mental health are also a determining factor, optimism plays a particularly relevant role, which can be understood as a psychological resilience strategy in the face of minority stress, particularly to the distal factors [27,55]. Optimism is perceived as the tendency to anticipate positive outcomes in the future [61,62] and can function as an internal force of protection and perseverance, especially in contexts marked by discrimination, institutional invisibility, or fragile social support [27,55]. In this sense, optimism in LGB students may not only be a dispositional characteristic but also an active resource that enables them to face adversity without losing hope, thereby contributing directly to their well-being [60,61,63].

It was also found that, in both groups of students, higher levels of motivation were significant predictors of well-being. However, academic performance was not found to be a predictor of well-being in LGB students. These results confirm the importance of the intention to engage in academic dynamics; motivation is often associated with greater involvement in tasks, greater persistence in the face of difficulties, and a perception of competence and autonomy, elements that contribute directly to subjective well-being [64]. However, the loss of predictive power of academic performance compared to the other predictor variables in LGB youth may reflect that, for this group, psychosocial factors such as social support, acceptance, and identity have a greater weight in the perception of well-being than academic performance itself [60,65,66].

Further, slightly higher levels of well-being and motivation were observed among heterosexual students. This difference, although subtle, may highlight the effect of contextual, emotional, and relational factors. The feeling of belonging, the perception of safety, and institutional recognition are key elements that directly influence subjective well-being and motivation levels [65,67]. On the other hand, LGB students continue to report less inclusive general institutional climates, characterized by microaggressions, curricular and social invisibility [17,68]. The lower motivation observed in LGB students can be interpreted as a reflection of contextual barriers that hinder the authentic expression of the self. Additionally, experiencing social exclusion or identity insecurity can impact academic self-efficacy, limiting the perception of control and the usefulness of personal effort [69]. These experiences can translate into progressive demotivation and feelings of exclusion, which are associated with lower academic engagement and satisfaction [48,70].

This study had some limitations. Namely, the cross-sectional design, non-probabilistic sampling, and the use of self-report instruments, which may have been subject to social desirability bias. Although participation was voluntary, individuals interested in the topic of sexual diversity and mental health may also have been drawn to participate in the study. The fact that the study was conducted exclusively in the context of Portuguese higher education limits the generalizability of the results to other sociocultural contexts. Another limitation was the use of a single-item to measure motivation and academic performance; similarly, the measure of experiential well-being showed modest internal consistency. Regarding future studies, the urgent need to promote inclusive, safe, and affirmative educational environments is highlighted, especially for students of non-heteronormative sexual orientations, as the data reinforce the importance of social support and peer connection. The development of projects that encourage initiatives adapted to the academic context, such as LGB peer mentoring programs and belonging groups, is pertinent; these can promote a sense of belonging, reduce isolation, and foster academic motivation [71].

Ultimately, this study presents both theoretical and practical implications. As for the theoretical implication, the study reinforces partially the minority stress model, acknowledging that LGB students experience lower levels of optimism, even when their academic performance is higher than that of their heterosexual peers [27]. This result suggests that it is essential to address not only the adverse effects of minority stress but also to foster the development of compensatory strategies that lead to academic success. Second, the results indicate that optimism is an important predictor of well-being among LGB students, while mental health is the main predictor in heterosexual students. This result theoretically contributes to resilience models in higher education contexts, suggesting that optimism and hope-based interventions may be crucial for understanding and supporting sexual minorities [72].

As such, universities can benefit from these results by developing specific psychological interventions that address the mental health and well-being of LGB students, including, for example, workshops directed to promote resilience, individual and group psychological support, which ultimately can help to reduce the impact of minority stress and increase the feeling of belonging. Considering that LGB students with higher optimism also exhibit higher well-being, practical interventions may help develop optimism and motivation, such as positive mindset training and mentoring activities [60]. These findings reinforce the importance of investigating not only the risks but also the protective factors (such as optimism, self-efficacy, and community) that promote resilience among LGB students, showing that a well-being-centred approach, rather than focusing solely on vulnerabilities, is essential [73,74,75].

## 5. Conclusions

This study highlighted relevant differences in mental health and predictors of well-being between heterosexual and LGB university students. The results confirm that LGB students exhibit higher levels of mental health and academic performance than heterosexual students, possibly reflecting adaptive strategies in the face of adverse contexts, such as lower levels of optimism, consistent with the minority stress model. While mental health emerged as the main predictor of well-being among heterosexual students, in LGB students’ well-being was explained by both mental health and optimism, highlighting the role of the latter as a psychological resource for resilience. Motivation proved to be a significant predictor of well-being in both groups, although academic performance did not directly contribute to the well-being of LGB students. Overall, the results highlight the need for more inclusive university environments and psychological interventions focused on promoting optimism, motivation, and social support, in order to reduce the impact of minority stress and promote the well-being and academic retention of LGB students.

## Figures and Tables

**Table 1:** Sample characteristics according to sexual orientation.

		Heterosexual	LGB
Characteristics		*N* = 129 *n* (*%*)	*N* = 156 *n* (*%*)
Age			
	*M* (*SD*)	27.73 (10.33)	24.48 (6.83)
Gender			
	Woman	80 (62)	102 (65.3)
	Man	49 (38)	50 (34.6)
Area of Residence			
	Mainland Portugal	96 (74.4)	117 (75.0)
	Islands	33 (25.6)	39 (25.0)
Marital Status			
	Single	84 (65.1)	114 (73.1)
	Married/Common-law marriage	37 (28.7)	14 (8.9)
	Widowed/Divorced/Other	6 (4.7)	28 (18)
Geographic Area of Study			
	Central Region	32 (24.8)	51 (32.7)
	Lisbon Region	29 (22.5)	31 (19.9)
	Islands	27 (20.9)	32 (20.5)
	North Region	19 (14.7)	16 (10.3)
	South Region	15 (11.6)	19 (12.2)
	Inland/Alentejo Region	3 (2.3)	6 (3.8)
Current Study Cycle			
	Undergraduate degree	80 (62)	95 (60.9)
	Master’s degree	41 (31.8)	54 (34.6)
	Doctorate degree	8 (6.2)	7 (4.5)
Current year of study			
	1st year of undergraduate degree	28 (21.7)	23 (14.7)
	2nd year of undergraduate degree	33 (25.6)	43 (27.6)
	3rd year of undergraduate degree	20 (15.5)	31 (19.9)
	1st year of master’s degree	16 (12.4)	15 (9.6)
	2nd year of master’s degree	11 (8.5)	20 (12.8)
	1st/2nd/3rd year of doctorate degree	9 (7)	4 (2.6)
Course of study			
	Health/Life Sciences/Sports sciences	33 (25.6)	35 (22.4)
	Education sciences/Social and behavioral sciences/Arts	29 (22.5)	48 (30.8)
	Business sciences/Law	25 (19.4)	17 (10.9)
	Engineering and related techniques/Mathematics/Computer Science	20 (15.5)	3 (1.9)

Note: The table does not include missing values.

**Table 2:** Correlations between variables according to sexual orientation.

	1	2	3	4	5
1. Well-being	-	0.153	−0.582 **	0.465 **	0.351 **
2. Academic performance	0.379 **	-	−0.134	0.211 *	0.134
3. Mental health	−0.754 **	−0.349 **	-	−0.362 **	−0.282 **
4. Optimism	0.740 **	0.307 **	−0.692 **	-	0.476 **
5. Motivation	0.607 **	0.385 **	−0.514 **	0.511 **	-

* *p* < 0.05; ** *p* < 0.01. Note: Correlations below the diagonal refer to the LGB population, and correlations above the diagonal refer to the heterosexual population.

**Table 3:** Descriptive data and hypothesis testing of the variables under study according to sexual orientation.

	Heterosexual			LGB			
	n *	*M*	*SD*	n *	*M*	*SD*	Test Statistic; *p* Value
Well-being	127	46.13	9.02	154	44.01	12.74	*t*(279) = 1.674 *p* = 0.091
Academic performance	115	13.84	4.38	141	14.93	2.83	*t*(254) = −2.039 *p* = 0.044
Mental health	122	44.28	16.91	147	38.73	17.49	*t*(267) = −2.604 *p* = 0.014
Optimism	127	16.19	3.17	155	14.57	3.56	*t*(280) = 3.869 *p* < 0.001
Motivation	128	3.61	1.12	155	3.37	1.17	*t*(281) *=* 1.709 *p* = 0.084

Note: *M =* mean; *SD* = standard deviation; *t* = *t*-test for independent samples; * Sample sizes vary across variables due to missing data.

**Table 4:** Linear Regression for well-being according to sexual orientation.

	Heterosexual (*N* = 115)				LGB (*N* = 141)			
*N* = 256	*B* (95% CI)	*E*	*β*	*t*	*B* (95% CI)	*E*	*β*	*t*
Academic performance	0.577 (0.244–0.890)	0.161	0.281	3.528 ***	0.231 (−0.242–0.719)	0.248	0.055	0.964
Mental Health	−0.265 (−0.356–−0.178)	0.046	−0.484	−5.789 ***	−0.262 (−0.366–−0.158)	0.056	−0.357	−4.899 ***
Optimism	0.760 (0.236–1.295)	0.271	0.275	2.891 **	1.371 (0.833–1.931)	0.268	0.361	5.048 ***
Motivation	1.012 (−0.412–2.427)	0.707	0.123	1.422 *	2.327 (1.074–3.569)	0.627	0.231	3.678 ***

* *p* < 0.05; ** *p* < 0.01; *** *p* < 0.001. Note: * Regression analyses were conducted using listwise deletion.

## Data Availability

The raw data supporting the conclusions of this article will be made available by the authors on request.
